# In situ modulation of intestinal organoid epithelial curvature through photoinduced viscoelasticity directs crypt morphogenesis

**DOI:** 10.1126/sciadv.add5668

**Published:** 2023-01-20

**Authors:** F. Max Yavitt, Bruce E. Kirkpatrick, Michael R. Blatchley, Kelly F. Speckl, Erfan Mohagheghian, Radu Moldovan, Ning Wang, Peter J. Dempsey, Kristi S. Anseth

**Affiliations:** ^1^ Department of Chemical and Biological Engineering, University of Colorado Boulder, Boulder, CO 80309, USA.; ^2^ BioFrontiers Institute, University of Colorado Boulder, Boulder, CO 80309, USA.; ^3^ Medical Scientist Training Program, University of Colorado Anschutz Medical Campus, Aurora, CO 80045, USA.; ^4^ Department of Mechanical Science and Engineering, The Grainger College of Engineering, University of Illinois at Urbana-Champaign, Urbana, IL 61801, USA.; ^5^ Advanced Light Microscopy Core Facility, University of Colorado Anschutz Medical Campus, Aurora, CO 80045, USA.; ^6^ Section of Developmental Biology, Department of Pediatrics, University of Colorado, Denver, CO 80204, USA.

## Abstract

Spatiotemporally coordinated transformations in epithelial curvature are necessary to generate crypt-villus structures during intestinal development. However, the temporal regulation of mechanotransduction pathways that drive crypt morphogenesis remains understudied. Intestinal organoids have proven useful to study crypt morphogenesis in vitro, yet the reliance on static culture scaffolds limits the ability to assess the temporal effects of changing curvature. Here, a photoinduced hydrogel cross-link exchange reaction is used to spatiotemporally alter epithelial curvature and study how dynamic changes in curvature influence mechanotransduction pathways to instruct crypt morphogenesis. Photopatterned curvature increased membrane tension and depolarization, which was required for subsequent nuclear localization of yes-associated protein 1 (YAP) observed 24 hours following curvature change. Curvature-directed crypt morphogenesis only occurred following a delay in the induction of differentiation that coincided with the delay in spatially restricted YAP localization, indicating that dynamic changes in curvature initiate epithelial curvature–dependent mechanotransduction pathways that temporally regulate crypt morphogenesis.

## Teaser

Spatiotemporal patterning of curvature in intestinal organoids activates mechanotransduction pathways that regulate crypt formation.

## INTRODUCTION

The mammalian intestine is a highly complex and dynamic organ that serves a variety of essential functions, including the absorption of nutrients from digested foods. To aid in this function, the intestinal epithelium forms distinctive finger-like protrusions into the lumen, called villi, that maximize the surface area of the intestine and enhance its absorptive capacity (*1*). Intestinal crypts are invaginations located between villi that house the secretory Paneth cells and leucine-rich repeat containing G protein–coupled receptor 5 (Lgr5)–positive intestinal stem cells (ISCs). ISCs are responsible for replenishing the various absorptive and secretory cell types that maintain intestinal function, while Paneth cells help to support ISC maintenance through cell-cell interactions and secreted signals (*2*). While the crypt niche is essential for maintaining the ISC population and thus intestinal function, the mechanisms that direct and initiate crypt morphogenesis remain incompletely understood [as reviewed by Kwon *et al*. (*3*)].

Intestinal organoids are powerful in vitro models that recapitulate the structure and function of the intestinal epithelium, enabling investigations into the mechanisms that regulate crypt morphogenesis (*4*). When encapsulated in a cell culture scaffold, a single ISC first proliferates to form a hollow spheroid, composed of a single-cell layer of ISCs. Upon the induction of differentiation conditions (i.e., removal of CHIR99021 and valproic acid), this spheroid undergoes a symmetry breaking event that results in differentiation to Paneth cells and produces crypt structures that protrude from the central body, forming a similar crypt-villus architecture as seen in vivo (*5*, *6*). Hartl *et al*. (*7*) tracked changes in cell morphology during intestinal organoid crypt development and found that spatially restricted changes in cell shape, specifically cellular elongation and apical narrowing in the region preceding crypt formation, were necessary to initiate crypt morphogenesis. Gjorevski *et al*. (*8*) exploited this geometric requirement by using micropatterned wells in soft hydrogels to force intestinal organoids into anisotropic geometries, where local differences in epithelial curvature directed the deterministic formation of crypts. It was shown that changes in tissue curvature, and the associated cellular shape and packing, induced spatial differences in yes-associated protein 1 (YAP) localization. Cell shape generates mechanical tension that is transmitted through cytoskeletal components to open nuclear pore complexes, enabling rapid translocation of YAP into the nucleus (*9*). In micropatterned epithelial monolayers, YAP nuclear translocation has also been shown to be spatially regulated by tension-dependent variations in resting membrane potential (*10*), as well as intranuclear distance and nuclear shape (*11*), mirroring findings relating to cell shape and nuclear packing within the crypt. Nuclear YAP binds to transcription factors to regulate gene expression (*12*), including the expression of delta-like canonical Notch ligand 1, a Notch ligand and known YAP target gene (*13*–*15*). In intestinal organoids, the symmetry breaking expression of Notch ligands confers secretory specification in YAP-activated cells that leads to the generation of the first Paneth cell and subsequent crypt formation (*16*).

While intestinal organoids have been used to investigate how epithelial curvature-induced YAP localization guides symmetry breaking events and crypt formation, greater knowledge of when and how cells commit to differentiation following changes in tissue curvature is needed to robustly characterize and engineer these living systems. Specifically, few studies have examined the spatiotemporal regulation of YAP localization following changes in tissue curvature. This lack in understanding is due, in part, to the reliance on static, user-defined surfaces that force epithelial cells into specific tissue geometries, which limits the study of dynamic processes that influence tissue morphogenesis. In particular, crypt morphogenesis in vivo is influenced by time-dependent changes in tissue geometry that occur during development and regeneration. For example, the formation of intestinal villi during intestinal development wrinkles the intestinal epithelium, altering the local curvature within specific intervillus domains that precede the location of intestinal crypts (*17*–*19*). However, these dynamic behaviors cannot be replicated in vitro using materials with static properties. Hence, little is known about how dynamic changes in curvature influence cell signaling and crypt formation.

In this work, we sought to develop a material platform that enables spatiotemporal control over tissue curvature to assess the role of dynamic cell shape changes on crypt formation. Previous work using photodegraded channels to locally guide crypt formation in intestinal organoids suggested that temporally dependent changes in cellular shape and nuclear spacing initiate crypt formation (*8*). However, this work relied on local degradation of the hydrogel, which is known to affect crypt development (*20*, *21*). Meanwhile, photomediated reactions that spatiotemporally control hydrogel viscoelasticity have been used to locally alter the shape of mesenchymal stem cells without changing hydrogel stiffness to decouple the two phenomena (*22*). Here, we use a photoinduced allyl sulfide exchange reaction to generate changes in hydrogel viscoelasticity, thereby locally and controllably deforming intestinal organoid epithelial shape to reveal a time-delayed change in YAP localization in response to cell shape modulation and the associated increase in mechanical tension and depolarization of the epithelial resting membrane potential. By varying the timing of the introduction of differentiation cues, we show that this delayed localization of YAP is necessary to instruct the cell shape–dependent patterning of crypt morphogenesis.

## RESULTS

### Allyl sulfide–mediated bond rearrangement facilitates photoinduced viscoelasticity

**Fig. 1.**
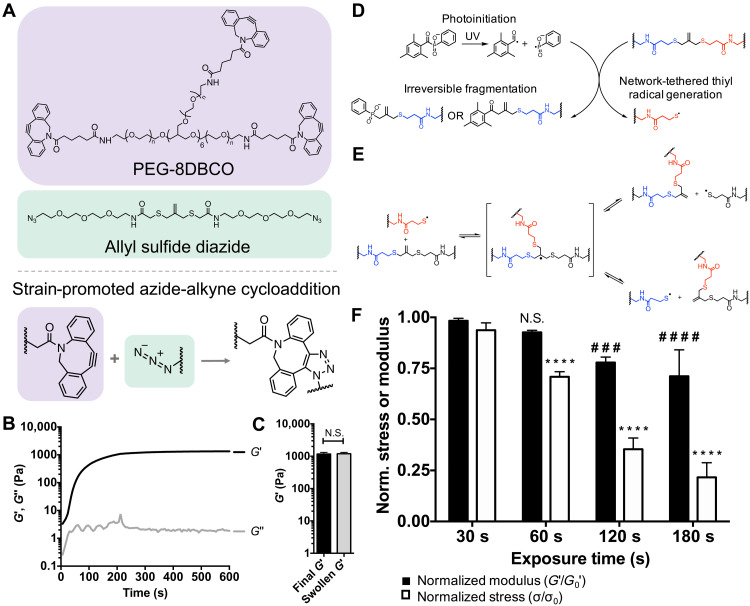
Hydrogel formation and photoinduced viscoelastic response. (**A**) Chemical structures of 8-arm poly(ethylene glycol) dibenzylcyclooctyne (PEG-8DBCO) and allyl sulfide diazide, which react through strain-promoted azide-alkyne cycloaddition (SPAAC) to form a hydrogel. (**B**) The shear storage modulus (*G*′; black) increased during gelation, reaching 90% of the plateau values at 236 ± 12 s, while the shear loss modulus (*G*″; gray) remains constant. Rheological plot shows representative trace. (**C**) Hydrogels achieved a final storage modulus of 1170 ± 150 Pa upon gelation, with no significant difference upon equilibration. Significance was determined by *t* test, *N* = 4 gels, means ± SD. (**D**) Incident ultraviolet (UV) light generates radicals from soluble lithium phenyl-2,4,6-trimethylbenzoylphosphinate (LAP) that react with allyl sulfide cross-links to generate network tethered thiyl radical species through irreversible fragmentation. (**E**) Network-tethered thiyl radical species then react with other allyl sulfides in an exchange reaction, regenerating an allyl sulfide cross-link and a network-tethered thiyl radical species, which can then participate in other exchange reactions. (**F**) The normalized modulus (black) and stress (white) of allyl sulfide cross-linked hydrogels decreased following increasing exposure times 405 nm light at 10 mW cm^−2^. Each sample was normalized to the modulus or stress value before irradiation. Significance was determined by two-way analysis of variance (ANOVA) with Tukey’s multiple comparison test; number sign (#) denotes significance compared to a 30-s normalized modulus condition, and asterisk (*) denotes significance compared to a 30-s normalized stress condition; ###*P* < 0.001; #### and ****P < 0.0001; *N* = 3 gels per condition, means ± SD. N.S., not significant.

With the intention of using light to modify epithelial shape in situ, we sought to design a hydrogel platform containing photosensitive allyl sulfide groups that also permitted three-dimensional (3D) encapsulation of intestinal organoids. Cross-linked hydrogels were formed through a strain-promoted azide-alkyne cycloaddition (SPAAC) reaction between 8-arm poly(ethylene glycol) dibenzylcyclooctyne (PEG-8DBCO) and allyl sulfide bis(PEG3-azide) (Fig. 1A). The gelation of this SPAAC hydrogel chemistry occurs rapidly in solution at physiological conditions and has been shown previously to be cytocompatible for 3D encapsulation of intestinal organoids (*21*, *23*, *24*). Rheological characterization of gelation indicated that the gel point, estimated by the crossover of the storage (*G*′) and loss (*G*″) moduli, occurred within 30 s, while 90% of the final *G*′ was achieved in 236 ± 12 s (Fig. 1B). Following gelation, *G*′ reached a plateau modulus of 1170 ± 150 Pa, which did not significantly change after gels were swollen to equilibrium in neutral phosphate-buffered saline (PBS; Fig. 1C). The final equilibrated *G*′ was chosen on the basis of previous work that defined the optimal range of modulus values for intestinal organoid formation under stem-inducing conditions (*G*′ = ~1.3 kPa) in synthetic hydrogels (*20*, *23*). Equilibrated hydrogels display no frequency dependence in the absence of light (fig. S1A).

Incorporation of the ally sulfide moiety within the network enables photoinduced, on-demand changes in hydrogel viscoelasticity. In this approach, ultraviolet (UV) light irradiation generates radicals from soluble photoinitiators that react with pendant alkene groups of the allyl sulfide cross-links, resulting in cleavage of the allyl sulfide to generate network-tethered thiyl radicals (Fig. 1D). These radical species then react with other allyl sulfide cross-links through a reversible addition fragmentation chain transfer reaction, generating new tethered thiyl radicals that can propagate and cause subsequent exchange reactions (Fig. 1E). Exchange reactions between network tethered reactive groups lead to bond rearrangement but do not significantly alter cross-link density, therefore temporarily increasing the hydrogel loss modulus (*G*″) while causing a minimal decrease to the storage modulus during irradiation (*G*′). This mechanism of bond rearrangement is different than what has been reported in other work using allyl sulfide hydrogels (*21*, *23*, *25*), which rely on cleavage of the allyl sulfide cross-link using a soluble monofunctional thiol species to strictly soften hydrogels (i.e., reduction in *G*′). To assess photoinduced viscoelasticity, hydrogels were equilibrated with 1 mM of the photoinitiator lithium phenyl-2,4,6-trimethylbenzoylphosphinate (LAP), and a rheometer with a light curing accessory was used to track in situ changes in *G*′, *G*″, and stress (σ) during irradiation (10 mW cm^−2^ of a 405-nm light). Because this reaction is driven by photoinduced radical generation, bond rearrangement only occurs for the duration of light exposure, providing temporal control over photoinduced bond rearrangement. *G*″ monotonically increases during irradiation, indicating increased viscoelasticity, and immediately decreases following shuttering of the light (fig. S1B). In addition, the normalized stress decreases throughout the duration of irradiation (fig. S1C). Figure 1F shows the normalized *G*′ and σ following increasing exposure times. At short exposure times of 30 and 60 s, the normalized σ significantly decreases, while there is no change in the normalized *G*′. However, at longer exposure times of 120 and 180 s, there is a significant decrease in both the normalized *G*′ and σ. Longer exposure times generate a higher concentration of LAP radicals, which subsequently consume more allyl sulfide cross-links through irreversible cleavage events to significantly degrade the network (fig. S2A). Despite the dependency of the rate of photoinitiation on the incident light intensity, it was found that the change in matrix properties was dependent on the total light dose (intensity × time), rather than the light intensity, indicating that concentration of radicals was still relatively low compared to the concentration of allyl sulfide cross-links (fig. S2B). These results indicate that bond rearrangement occurs in the absence of degradation at lower light doses, providing a mechanism for photoinduced bond rearrangement with minimal changes to the local hydrogel storage modulus.

### Magnetic rotational spectroscopy reveals minimal change in *G*′ following spatial photopatterning

**Fig. 2.**
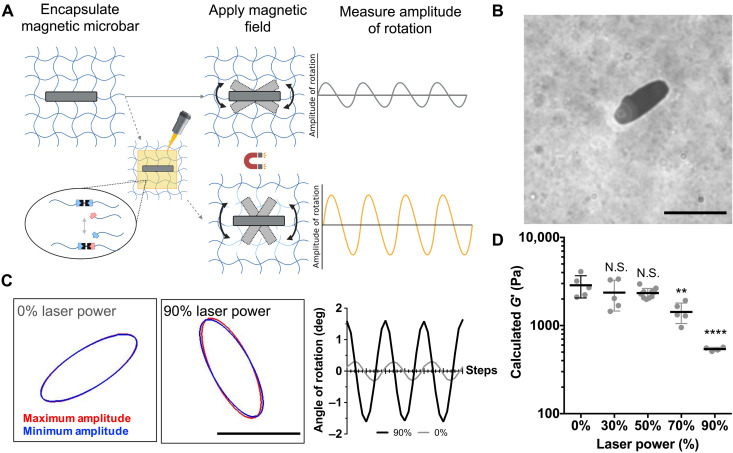
Microrheology within photopatterned regions. (**A**) Magnetic microbars were encapsulated within an allyl sulfide hydrogel network, and an oscillating magnetic field was applied, causing the microbars to rotate. Images of microbar oscillations were obtained and used to determine the local rheological properties, as the amplitude of rotation is a measure of the local hydrogel modulus. A laser scanning confocal microscope was then used to photopattern regions for photoinduced viscoelasticity around the encapsulated microbars. In this case, degradation from the allyl sulfide bond exchange reaction causes an increase in the amplitude. (**B**) Encapsulated microbars were 31.6 ± 1.6 μm in length and 12.6 ± 1.4 μm in width. Scale bar, 30 μm. (**C**) Microbars within regions photopatterned with 0 (left) and 90% (right) laser power oscillated between a minimum (blue) and maximum (red) amplitude. The angle of rotation is shown for microbars oscillating following 0% (gray) and 90% (black) laser power to visualize the amplitude of rotation. (**D**) The amplitude of microbar rotation was used to calculate *G*′ (see the Supplementary Materials), which decreased at higher light doses, indicating local hydrogel degradation due to irreversible fragmentation events. Significance was determined by one-way ANOVA with Tukey’s multiple comparison test; **P < 0.01; ****P < 0.0001; *N* = 5, 5, 10, 5, and 4 microbars for 0, 30, 50, 70, and 90% laser power, respectively. Black bar denotes average ± SD, while gray points denote measurements from individual microbars.

While rheology was used to characterize the bulk changes in viscoelastic behavior in response to flood exposure to low doses of UV light, we next sought to assess local changes in rheological properties in response to spatially patterned light. Here, a laser scanning confocal microscope was used to photopattern regions of induced viscoelasticity. A laser delivers a much higher light dose over a shorter time, compared to a UV lamp, which influences the concentration of photoinitiated radicals and thus the kinetics of the allyl sulfide bond rearrangement reaction (*21*). To characterize local changes in the hydrogel properties and identify photopatterning conditions that do not affect *G*′, we used magnetic rotational spectroscopy (*26*, *27*), a microrheological technique, to monitor *G*′ in photopatterned regions (Fig. 2A). Magnetic microbars measuring 31.6 ± 1.6 μm in length and 12.6 ± 1.4 μm in width were first encapsulated within allyl sulfide hydrogels (Fig. 2B). Hydrogels were then equilibrated with 1 mM LAP, and a laser scanning confocal microscope was used to apply light (405 nm, 10 to 90% laser power) to square patterned regions in a single plane surrounding encapsulated microbars to initiate bond rearrangement. Because of laser light traveling through the material, irradiation of a single plane generates a roughly uniform pattern throughout the depth of the hydrogel (fig. S3, A and B). An oscillating magnetic field was applied, causing the microbars to rotate (Fig. 2C), from which the amplitude of rotation was used to calculate the shear storage modulus within the photopatterned regions (*28*). Results show that *G*′ remains constant until a laser power of 70% is used, corresponding to a light dose of 4 J cm^−2^ (Fig. 2D). At this point, irreversible fragmentation from photocleaved LAP molecules led to degradation of the network that significantly decreased *G*′, as observed by an increase in the microbar rotation. This result is consistent with bulk rheological measurements, which showed increased degradation at higher light doses. In addition, these data motivate the selection of 30% laser power, corresponding to a light dose of 1.80 J cm^−2^, as appropriate photopatterning conditions to induce viscoelastic changes in cell laden hydrogels with minimal reduction in *G*′. This light dose is an order of magnitude lower than doses reported to cause damage to cells in literature (*29*).

### Photopatterning of viscoelasticity enables control over epithelial curvature

**Fig. 3.**
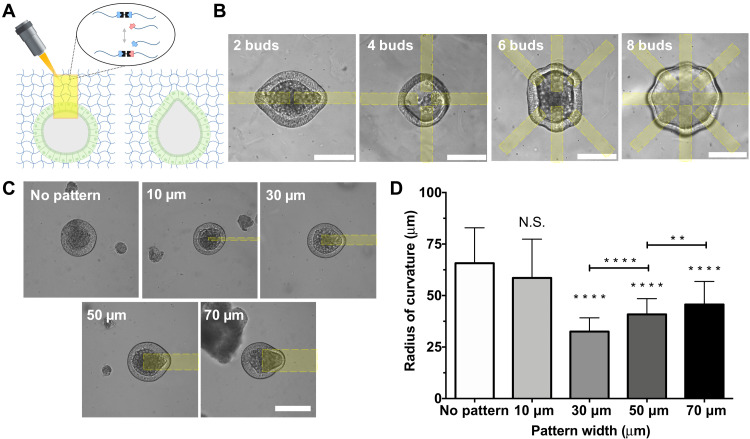
Photopatterned viscoelasticity locally alters epithelial curvature. (**A**) Application of photopatterned light initiates local hydrogel bond rearrangement, deforming the epithelium within the patterned region. (**B**) Selection of pattern spacing can be used to generate up to eight independent deformations within a single organoid. Scale bars, 100 μm. (**C**) The width of the photopattern determines the width of epithelial curvature. (**D**) Epithelial curvature is unchanged by a 10-μm pattern but is decreased according to pattern width with 30-, 50-, and 70-μm patterns. Significance was determined by one-way ANOVA with Tukey’s multiple comparison test; **P < 0.01; ****P < 0.0001; *N* = 29, 29, 103, 121, and 121 organoids for no pattern and 10-, 30-, 50-, and 70-μm conditions, respectively. Bars denote average ± SD.

After quantifying the changes to the hydrogel mechanical properties as a function of light dose, selected conditions for the photoinduced viscoelasticity mechanism were used to introduce spatially defined deformations in intestinal organoid epithelia. Because the application of light affords spatial control over the material property changes, intestinal organoids were encapsulated within allyl sulfide hydrogels and equilibrated with photoinitiator, and a laser scanning confocal microscope was subsequently used to raster local regions of network bond rearrangements adjacent to encapsulated organoids (Fig. 3A). Intestinal organoids were uniformly distributed throughout the depth of hydrogel, such that light attenuation has an insignificant effect on the hydrogel properties (fig. S3C). Specifically, irradiation initiates the photoinduced bond rearrangement mechanism to relax a portion of the stress within the photopatterned regions, resulting in localized deformation of the epithelium. Under growth conditions, the physical expansion of encapsulated organoids is restricted by elastic confinement of the hydrogel network that generates local stresses. Relaxation of these stresses via the photoinduced bond rearrangement reaction leads to a physical deformation of the organoid epithelia that can be seen immediately after photopatterning (fig. S4). Once the light is shuttered, the bond rearrangement reaction is stopped, and the hydrogel returns to an elastic state to preserve the state of deformation. By controlling the spatial distribution of the photopatterned regions, the shape of the organoid epithelium can be controlled. Figure 3B shows the patterning of eight distinct bud-like regions within a single organoid. In addition, the width of the photopatterned regions ultimately determines the curvature of the deformation, with smaller patterned regions resulting in decreases in the radius of curvature (Fig. 3C). A 10-μm wide pattern did not generate a change in the epithelial curvature compared to unpatterned organoids. Presumably, the 10-μm pattern is too small to allow for deformation. Meanwhile, 30-, 50-, and 70-μm wide patterns generated deformations with radii of curvature of 32 ± 6.7, 40.8 ± 7.6, and 54.6 ± 11.2 μm, respectively, based on measured curvature values (Fig. 3D). In addition, the epithelial curvature was insensitive to the length of the photopatterned region (fig. S5). Collectively, these results demonstrated that the photopatterned region size can be used to tune the specific epithelial curvature.

### Regions of photopatterned curvature template and direct the formation of crypts

**Fig. 4.**
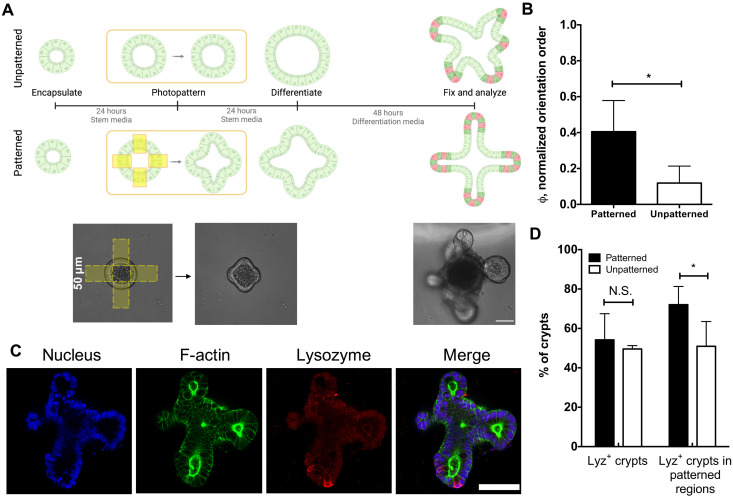
Photopatterned changes in epithelial curvature template crypt formation. (**A**) Organoids were encapsulated into hydrogels and allowed to expand for 24 hours before applying photopatterns. The “unpatterned” condition did not receive any light dose. After 24 hours, patterned and unpatterned organoids were exposed to differentiation conditions, and crypt growth was assessed 48 hours later. Scale bar, 50 μm. (**B**) The angles of crypt formation were used to generate an orientation order parameter, which is higher for patterned organoids (black) compared to unpatterned (white) organoids, indicating that crypt formation is directed and templated by patterned epithelial shape. Significance was determined by *t* test; *P < 0.05; *N* = 4 and 3 gels for the patterned and unpatterned conditions, respectively. Bars denote average ± SD. (**C**) Immunostaining of differentiated organoids for nuclei (blue), F-actin (green), and lysozyme (red), a marker for Paneth cells, revealed Paneth cells confined to crypt ends. Scale bar, 50 μm. (**D**) Analysis of lysozyme distribution in patterned (black) and unpatterned (white) organoids revealed that 54 ± 13% and 49 ± 2% of all crypts contained lysozyme-positive cells in patterned and unpatterned organoids, respectively, while 72 ± 9% and 50 ± 12% of crypts in patterned regions contained lysozyme-positive cells in patterned and unpatterned organoids, respectively. Significance was determined by one-way ANOVA with Tukey’s multiple comparison test; *P < 0.05; *N* = 4 gels. Bars denote average ± SD.

To determine whether photopatterned curvature changes could direct crypt formation, four evenly spaced photopatterned regions (50 μm in width) were first applied to regions adjacent to encapsulated organoids, deforming initially spherical organoids into cross-shape organoids. Twenty-four hours after patterning of the epithelial curvature, organoids were then exposed to differentiation conditions (bulk hydrogel degradation and removal of CHIR99021 and valproic acid) to initiate crypt formation (Fig. 4A) (*21*). Before differentiation, the application of photopatterned viscoelasticity did not impede isometric expansion in stem growth conditions (fig. S6). The angle of the resulting crypt formation was measured relative to the initial patterned regions and used to calculate the orientation order parameter, which is a measure of the spatial entropy of the system (see fig. S7 and Materials and Methods for further explanation) (*30*). Results in Fig. 4B reveal that crypts originating from shape patterned organoids display a higher orientation order parameter, compared to unpatterned spherical organoids, meaning that crypts form more often from regions of photopatterned shape change and that the directionality of crypt formation is more aligned. In addition, immunostaining confirmed the presence of lysozyme, a marker for Paneth cells, residing within the crypt regions (Fig. 4C). An equal number of crypts contained lysozyme positive cells in patterned (54 ± 13%) and unpatterned (49 ± 2%) organoids, while more lysozyme positive crypts existed specifically within patterned regions of patterned organoids (72 ± 9%), compared to unpatterned organoids binned in a similar manner (50 ± 12%) (Fig. 4D). From these results, we posit that the photoinduced changes to the local stress distribution prime patterned regions within the organoid for crypt formation through cell shape–induced mechanotransduction.

### Photopatterned curvature results in increased tension and membrane depolarization

**Fig. 5.**
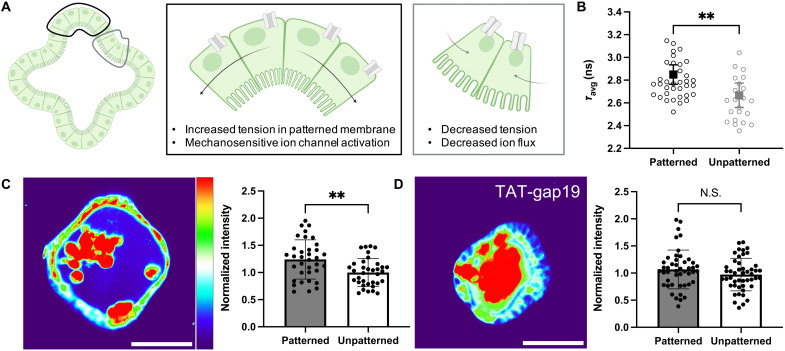
Induced curvature alters membrane tension and resting membrane potential. (**A**) Cells within regions of photoinduced shape change (black outline) are deformed, increasing membrane tension and stretch-sensitive ion channel activation compared to unpatterned regions (gray outline). (**B**) Fluorescence lifetime of the membrane tension probe FlipperTR increases in patterned regions, indicating higher membrane tension. *N* = 9 patterned (36 regions) versus 6 unpatterned (22 regions) organoids. (**C**) Resting membrane potential, as measured by fluorescence intensity of the voltage sensitive dye DiSBAC_2_ (*3*), is increased in patterned regions compared to unpatterned regions, suggesting that membrane depolarization colocalizes with increased tension. *N* = 35 paired patterned versus unpatterned regions from nine organoids. (**D**) Inhibition of connexin hemichannels with TAT-gap19 diminishes bioelectric patterning observed at baseline. *N* = 46 paired patterned versus unpatterned regions from 12 organoids. Scale bars, 50 μm. Significance was determined by unpaired *t* tests with Welch’s correction; means ± SD are shown.

To understand how photoinduced curvature locally activates mechanotransduction pathways, we first sought to elucidate the change in mechanical tension and resting membrane potential. Recent work using micropatterned mammary epithelial monolayers demonstrated that regions of increased tension also showed greater resting membrane potential (depolarization) and subsequent nuclear localization of YAP that drove corresponding patterns of proliferation. Treatment with the connexin hemichannel inhibitor, TAT-gap19, blocked ion channels to diminish the spatially localized patterning in membrane potential and YAP localization (*10*). Appreciating the potential relationship between epithelial curvature, membrane tension, and localized activation of stretch-sensitive ion channels (Fig. 5A), we first aimed to determine whether membrane tension was affected by photoinduced shape change. Within 2 to 4 hours of photopatterning, organoids were stained with the membrane tension reporter dye Flipper-TR (*31*) for fluorescence lifetime imaging microscopy (FLIM) analysis. FlipperTR localizes to the cell membrane and has a fluorescence lifetime on the order of nanoseconds, which increases or decreases when the membrane is in tension or compression, respectively. Using a phasor approach to frequency domain FLIM analysis (fig. S8A) (*32*), we measured an increase in average fluorescence lifetime of 182 ± 52 ps in patterned regions compared to unpatterned regions (Fig. 5B). Having established that photopatterning induced curvature results in greater membrane tension, we next investigated the effect of curvature on the resting membrane potential, which is dependent on the membrane tension (*10*) and has been highlighted as a possible symmetry breaking cue in homogenous tissues (*33*) and as an understudied element of intestinal biomechanics (*34*). Using the anionic dye bis-(1,3-diethylthiobarbituric acid) trimethine oxonol (DiSBAC_2_) (*3*), which is concentrated in regions of the lipid bilayer with increasing membrane depolarization, we quantified fluorescence intensity in patterned versus unpatterned regions of the membrane and found significant depolarization in regions of high curvature and tension (Fig. 5C and fig. S7B). Treating organoids with TAT-gap19 for 5 hours after photopatterning resulted in elimination of this clear patterning of the resting membrane potential (Fig. 5D), implicating connexin hemichannels in the maintenance of resting membrane potential patterns in intestinal organoids. Together, these results show that photoinduced shape change results in spatial distributions of increased tension and membrane depolarization.

### YAP localization is delayed following curvature patterning

**Fig. 6.**
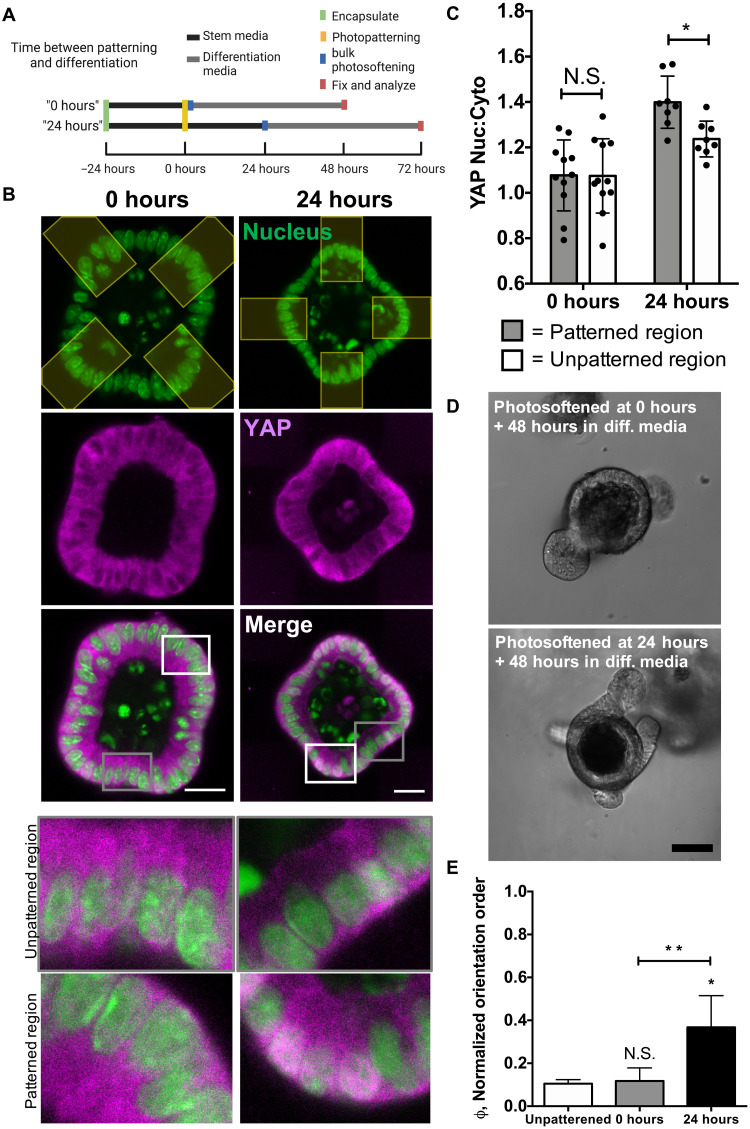
Photopatterned shape change precedes downstream YAP expression to direct cell fate. (**A**) Organoids were encapsulated 24 hours before photopatterning to induce epithelial curvature at *t* = 0 hours. Photopatterned organoids were exposed to differentiation conditions immediately after photopatterning for the “0-hour” condition and 24 hours after photopatterning for the “24-hour” condition. Organoids were fixed 48 hours after differentiating. (**B**) YAP (magenta) and nuclei (green) in organoids, either 0 (left) or 24 (right) hours following photopatterning of a cross shape. The photopatterned region is pseudo-colored in yellow. Scale bars, 30 μm. Insets show more detail of the unpatterned (top) and patterned regions (bottom) shown in the merged images. (**C**) YAP/TAZ nuclear to cytoplasmic ratio increased in patterned regions of organoids after 24 hours after patterning but remains unchanged 0 hours after patterning. Significance was determined by two-way ANOVA with Tukey’s multiple comparison test; *P < 0.05. *N* = 11 organoids from two gels for the 0-hour condition, *N* = 8 organoids from two gels for the 24-hour condition. (**D**) Organoids differentiated following patterning produce crypts that align with patterned shape changes after 24 hours. Scale bar, 50 μm. (**E**) Organoids differentiated 0 hours (gray) after patterning produce crypts that align similar to unpatterned organoids (white), while organoids differentiated 24 hours (black) after patterning produce crypts that align with patterned regions. Significance was determined by one-way ANOVA with Tukey’s multiple comparison test; *P < 0.05; **P < 0.01; *N* = 3, 5, and 4 gels for unpatterned, 0 hours, and 24 hours, respectively. Bars denote average ± SD.

With the understanding that dynamic changes in organoid curvature (i.e., from spherical to the induction of buds) influence the resting membrane potential and the subsequent evolution of differentiation and crypt formation, we next sought to monitor the spatial and temporal distribution of YAP expression after photopatterning and the effect of YAP localization on crypt formation. Recent literature has suggested tissue curvature–induced changes in YAP expression are a driving force in directing crypt formation from ISCs (*8*); however, these experiments were conducted using geometrically defined templates that generated static organoid structures before differentiation. Hence, we applied photopatterns to encapsulated organoids to induce changes in shape and then monitored the localization of YAP over time (Fig. 6A). Immediately after photopatterning (0 hours), YAP was evenly distributed between the nucleus and cytoplasm for cells located within both patterned and unpatterned regions. However, 24 hours after photopatterning, a higher nuclear to cytoplasmic ratio was observed in cells within patterned regions compared to cells residing in unpatterned regions of the organoids (Fig. 6, B and C). While the data show a relatively small increase in YAP nuclear-cytoplasmic localization, values of this magnitude have been reported elsewhere in literature for inducing patterned changes in YAP localization that result in localized proliferation and differentiation (*8*, *10*). This result suggests a temporal dependence on YAP localization, namely, that active changes in tissue curvature and cell shape precede changes in YAP localization before crypt formation. To test this hypothesis and correlate differences in YAP expression with crypt formation events, organoids were either differentiated immediately (0 hours) or 24 hours after patterning (Fig. 6, D and E). The orientation order parameter calculated for organoids differentiated 0 hours after patterning (0.12 ± 0.06) was not significantly different from that of unpatterned organoids (0.10 ± 0.02), indicating that patterning of epithelial shape does not influence crypt formation at early time points. Differentiating organoids at 24 hours after patterning of the tissue curvature resulted in a greater orientation order parameter (0.37 ± 0.14) compared to the unpatterned control. Because differences in YAP localization are not realized immediately after patterning of epithelial shape, these results support the notion that photoinduced change in tissue curvature leads to spatially altered YAP expression, which is a driving force for symmetry breaking and crypt formation (*14*). Moreover, treatment with TAT-gap19 resulted in a significant decrease in YAP nuclear to cytoplasmic ratio in patterned versus unpatterned regions (fig. S9), suggesting that supracellular patterns in tension and resting membrane potential may be necessary for programmed YAP localization and symmetry breaking in our 3D hydrogel system.

## DISCUSSION

During intestinal organoid development, local changes in epithelial cell shape are necessary to drive symmetry breaking events that lead to crypt formation, but there is little understanding of the role of epithelial curvature induced mechanotransduction in directing crypt development (*35*). Specifically, we were interested in understanding how mechanotransduction pathways are temporally regulated to influence symmetry breaking events. To answer this question, we sought to design materials that enabled spatiotemporal control over epithelial cell shape to study the progression of mechanotransduction pathways following active changes in tissue curvature. The photoinduced allyl sulfide bond rearrangement mechanism was originally used to mitigate polymerization-induced shrinkage stresses in polymer networks (*36*). This process was later exploited in allyl sulfide cross-linked hydrogels to spatiotemporally alter viscoelastic properties to probe the time-dependent cellular responses of hMSCs and to direct their cell shape (*22*). In this work, we highlight the ability of this photoinduced bond rearrangement mechanism to temporarily alter viscoelastic properties to direct epithelial shape. In addition, while recent literature has begun to investigate the role of matrix viscoelasticity on intestinal organoid development (*37*–*39*), the effects of temporary changes in viscoelasticity have yet to be explored. Here, the patterned application of the allyl sulfide bond rearrangement reaction to discrete regions of the hydrogel relaxes stresses built up by the expanding organoid, causing epithelial deformation into the patterned region to impart distinct and controllable curvature into the epithelium that persists to influence organoid crypt formation. This reaction occurs with minimal change to the hydrogel storage modulus, as elastic properties are known to be strong regulators of mechanotransduction and crypt formation in intestinal organoids (*20*, *21*). Figure 6C shows that photopatterned viscoelasticity alone is not enough to influence crypt formation, as only organoids differentiated 24 hours after patterning show crypt alignment with the photopatterned regions. If the photoinduced viscoelastic reaction was sufficient to degrade the network, then these patterned regions would influence crypt alignment. Instead, differentiating immediately following patterning results in no difference in the alignment of crypt emergence compared to unpatterned organoids (Fig. 6D). Hence, this platform enables minimization of the effects of hydrogel mechanics on crypt formation to isolate the role of tissue curvature on organoid development.

Following photopatterned changes in epithelial shape, differentiation was induced by photosoftening and altering medium composition to initiate crypt formation. It was found that crypts originated from regions of photopatterned shape change with higher frequency than unpatterned regions, indicating that epithelial shape can direct the location of crypt formation. Unperturbed organoid crypt morphogenesis generates a random distribution of crypt architectures, including the spacing and distribution of crypts, which contributes to culture heterogeneity and hinders the translational potential of organoid technologies (*40*). Instead, we show that control over tissue curvature can be used to template the location of crypt formation, providing an opportunity to increase the homogeneity of organoid culture. Other work has used various lithographic patterning methods to generate biomaterial scaffolds that mimic the crypt-villus architecture, forcing the development of more uniform structures in an organoid monolayer culture (*41*–*44*). However, few methods have been developed to direct organoid morphology within 3D encapsulated organoids.

With respect to epithelial tissue, the localization of YAP is strongly influenced by cell shape and tissue curvature (*9*, *11*). Previous studies have identified tissue curvature–induced YAP localization as a driving force for crypt morphogenesis. Gjorevski *et al*. (*8*) used lithographic microwells to confine intestinal organoids in elongated shapes to assess the effect of tissue curvature and cell packing on crypt morphogenesis. Regions of high curvature have higher nuclear packing that results in decreased nuclear YAP activation compared to regions with no curvature. This spatial difference in YAP activation initiates Notch ligand expression to confer Paneth cell fate specification and crypt formation. However, this strategy used static, nonadaptable materials that do not capture the dynamic changes in tissue curvature experienced in vivo during intestinal development nor enable investigations into the temporal regulation of these mechanotransduction programs. Recently, a self-rolling elastomer thin film was used to investigate how epithelial sheets dynamically respond to changes in tissue curvature (*45*), yet this curvature was not spatially restricted to recapitulate the local change in curvature seen in the formation of intervillus domains during intestinal development. Hence, there is little understanding of how cells respond to active changes in tissue curvature that occur during development.

Here, we show that photopatterned curvature in intestinal organoids elicits an increase in the YAP nuclear:cytoplasmic ratio that can be observed 24 hours after induced shape change and is regulated by a tension-induced gradient in resting membrane potential. This change in YAP localization was essential to template crypt formation, as photopatterned curvature had no effect on symmetry breaking before spatially restricted YAP localization. The presented photopatterning method enables spatiotemporal control over tissue geometry to more closely replicate active changes in tissue geometry seen during development that could provide insight into the role of mechanotransduction in the regulation of intestinal development. Laser nanosurgery experiments performed by Liberali and coworkers identified increased tension (as measured by recoil velocity of severed actin) in bulged and budded crypts (*4*). Because the allyl sulfide hydrogel system directly induces this increase in tension before any stochastic budding occurs, our results imply that internal feedback mechanisms likely regulate symmetry breaking events, allowing for cells to integrate information about their physical state to coordinate collective fate decisions (*46*).

In summary, we have used the allyl sulfide photoinduced bond rearrangement reaction to spatiotemporally modulate hydrogel viscoelasticity to control intestinal organoid epithelial shape with minimal change to the local storage modulus. Spatially localized deformations in epithelial shape induce membrane potential–dependent YAP nuclear translocation that template the formation of intestinal crypts. This platform enables control over crypt morphology to generate more homogenous multicellular architectures and enables investigations into the temporal regulation of mechanotransduction pathways following changes in cell shape, which could provide insight into the biomechanical processes that direct crypt morphogenesis during intestinal development and regeneration.

## MATERIALS AND METHODS

### Synthesis of PEG-8DBCO (40 kDa)

Eight-arm, 40-kDa PEG-8DBCO macromers were synthesized by coupling DBCO C6 acid (100 mg, 0.3 mmol; Click Chemistry Tools) to PEG-8 amine [0.5 g, 0.025 mmol, weight-average molecular weight (*M*_w_) = 20,000; JenKem USA] using (1-[bis(dimethylamino)methylene]-1H-1,2,3-triazolo[4,5-b]pyridinium 3-oxid hexafluorophosphate (121 mg, 0.32 mmol; ChemPep) as an activator and *N*,*N*-diisopropylethylamine as a base (0.175 ml, 1 mmol; Sigma-Aldrich). The reaction was purged with argon and allowed to proceed overnight. The reaction mixture was precipitated in cold diethyl ether (×3) to yield an off-white solid, which was collected by centrifugation, dissolved in water, dialyzed (8-kDa *M*_W_ cutoff; Spectrum Chemical) for 48 hours against deionized water, frozen, and lyophilized to yield the final product (93% yield, 80% functionalization by 1H nuclear magnetic resonance).

### Synthesis of allyl sulfide diazide

The allyl sulfide diazide cross-linker was synthesized as previously described (*18*).

### Characterization of hydrogel formation

Hydrogels containing allyl sulfide cross-links were formed with a SPAAC reaction by mixing stock solutions of 20 weight % (wt %) PEG-8DBCO in ice-cold PBS with allyl sulfide diazide in dimethyl sulfoxide (DMSO) to a final concentration of 4 wt % PEG and 2.4 mM allyl sulfide in PBS. The precursor solution was immediately vortexed for 5 s and placed as a 13-μl drop onto the bottom plate of a shear oscillatory rheometer (DHR-3, TA Instruments) fitted with an 8-mm parallel plate geometry. The storage and loss moduli during gelation were recorded using a strain of 5% and a frequency of 1 Hz. Once gelled, the hydrogel was equilibrated with PBS on the rheometer for 30 min, and the storage and loss moduli were again recorded at the same strain and frequency.

### Characterization of hydrogel degradation and stress relaxation

Hydrogels containing allyl sulfide cross-links were formed with SPAAC by mixing stock solutions of 20 wt % PEG-8DBCO in ice-cold PBS with allyl sulfide diazide in DMSO to a final concentration of 4 wt % PEG and 2.4 mM allyl sulfide in PBS. The precursor solutions were immediately vortexed for 5 s and placed as 23-μl drops between two, Sigmacote-treated glass slides separated by 0.5-mm spacers. The glass slides were clamped together and set aside to gel for 10 min. The fully formed gels were then placed in 500 μl of baths containing 1 mM LAP and 0.92 mM PEG3-azide in 100 mM Mops buffer (pH 7.4) to equilibrate for 20 min. Oscillatory rheology was performed on the gels using a TA Instruments DHR-3 rheometer with an 8-mm parallel plate geometry. The rheometer was fitted with an adaptor to allow for light exposure from a mercury arc lamp (OmniCure) fitted with a 400- to 500-nm band-pass filter. The storage and loss moduli were recorded at a strain of 5% and a frequency of 1 Hz before and after running a stress relaxation protocol. For the stress relaxation protocol, hydrogels were brought to a 10% strain and irradiated with light at 10 mW cm^−2^ for 30, 60, 120, or 180 s. The change in stress as well as the final and initial storage and loss moduli values were recorded.

### Calculation of LAP concentration

The theoretical photoinitiator consumption can be calculated by assuming first-order kinetics of the photolysis reaction (*47*)

$$\frac{d[\mathrm{LAP}]}{dt}=-k\;[\mathrm{LAP}]$$ (1)

where the rate constant, *k*, is defined by

$$k=\frac{\phi \;I_{o}\;\epsilon \;\lambda \;\ln(10)}{N_{A}\;h\;c}$$ (2)

where ϕ is the quantum yield of the initiator (assumed unity), *I*_o_ is the incident light intensity (10 mW cm^−2^), ε is the molar extinction coefficient (32 M^−1^ cm^−1^) (*48*) at the given wavelength λ (405 nm), *N*_A_ is Avogadro’s number, *h* is Planck’s constant, and *c* is the speed of light. Solving this gives *k* = 0.0025 s^−1^, and then, the LAP concentration can be calculated from the integrated rate equation

$$[\mathrm{LAP}]={\left[\mathrm{LAP}\right]}_{0}e^{-0.0025\;t}$$ (3)

### Fabrication of magnetic microbars

An array of ferromagnetic cobalt-platinum microbars was electroplated onto a silicon substrate using previously reported methods with some modifications (*49*). First, a 4-inch single side polished silicon wafer (Pure Wafer, San Jose, CA) was sputtered with a 45-nm Ti adhesion layer, followed by a 45-nm TiN diffusion barrier layer and a 80-nm Cu seed layer for electroplating (AJA ATC Orion 8 UVH, AJA International). Following the seed layer stack deposition, the microbar pattern was then transferred on the wafer by first spin coating the 10-μm AZ9260 positive photoresist (MicroChemicals Inc.) and subsequently exposing the photoresist with UV light (I line 365; EVG 620 Mask Aligner, EV Group) through the chromium photomask designed by AutoCAD (Autodesk Inc.). To remove any possible resist residues, the resulting wafer was treated with 150-W power oxygen plasma for 3 min (March Jupiter III). Electrodeposition was then carried out in a custom-made galvanostatic cell with 1 liter of electrolyte containing 0.025 M diamminedinitritoplatinum (II) solution [Pt(NH_3_)_2_(NO_2_)_2_] (MilliporeSigma), 0.1 M cobaltous sulfamate [Co(NH2)2(SO3)2] (Alfa Aesar), and 0.1 M ammonium citrate [(NH_4_)_2_C_6_H_6_O_7_] (MilliporeSigma) with no stirring and using Pt as the counter electrode. The CoPt was deposited by applying the current density of 50 mA cm^−2^ for 60 min corresponding to a thickness of 7 μm. Following the deposition, the CoPt was then annealed in high vacuum at 675°C in 30 min, resulting in phase transition from disordered A1 phase to the ordered L10 phase with high coercivity. Last, the microbars were removed from the wafer with brief sonication in water bath and collected in 5-ml centrifuge tube.

### Determination of hydrogel modulus by magnetic rotational spectroscopy

Magnetic microbars were encapsulated into ally sulfide hydrogels and equilibrated with 1 mM LAP in Mops buffer (100 mM, pH 7.4) for 15 min. The medium was removed, and a laser scanning confocal microscope was used to apply a 405-nm light (1 mW) of varying laser power with a pixel size of 290 nm and a dwell time of 5.12 μs to square regions of interest (ROIs) (100 μm by 100 μm) around single encapsulated microbars. Following photopatterning, hydrogels were placed into PBS. The hydrogels were then placed in in 2.9-T magnetization field of a Prisma magnetic resonance imaging (Siemens Healthineers) to permanently magnetize the encapsulated microbar along the *x* axis. Following magnetization, samples were placed in 3D magnetic twisting cytometry (*50*), and sinusoidal twisting field (25 G at 0.3 Hz) was applied along the *y* axis, resulting in the plane sinusoidal rotation of the microbar, and images of the rotating microbars were captured. Using Fiji [National Institutes of Health (NIH)], images of microbar oscillations were thresholded and masked and fit to ellipses to determine the angle of rotation. The shear storage modulus, *G*′, was determined by the following equation, adapted from (*51*)

$$G^{\prime }=\frac{M_{S}}{6\;\omega \;C(n)}\mu_{0}H_{A}$$ (4)

Here, *M*_s_ is the saturation magnetization for CoPt (6.5 × 105 A/m) (*52*), ω is the rotation angle determined by imaging, *C*(*n*) is a shape correction factor [9.09; calculated from (*28*)], and μ_0_*H*_A_ is the magnetic field strength (2.5 × 10^−3^ kg A^−1^ s^−2^).

### Crypt isolation and organoid culture

Murine small intestinal crypts were extracted from Lgr5-eGFP-IRES-CreERT2 mice as previously described (*20*). Isolated crypts were cultured as organoids by embedding in reduced growth factor Matrigel (Corning). Organoids were maintained in Advanced Dulbecco’s Modified Eagle’s Medium (DMEM)/F-12 (Invitrogen) containing N2 and B27 supplements (Thermo Fisher Scientific), GlutaMAX (Gibco), Hepes, and penicillin-streptomycin supplemented with epidermal growth factor (EGF; 50 ng/ml; R&D Systems), Noggin (100 ng/ml; PeproTech), R-spondin–conditioned medium (5% v/v; Organoid & Tissue Modeling Shared Resource, CU Anschutz), CHIR99021 (3 μM; Selleckchem), and valproic acid (1 mM; Sigma-Aldrich). “Basal medium” refers to Advanced DMEM/F-12 supplemented with N2 and B27 supplements, Hepes, and penicillin-streptomycin; while “complete medium” refers to basal medium with EGF, noggin, R-spondin, CHIR99021, and valproic acid (ENRCV). The medium was changed every 2 days, and organoids were passaged every 4 days. The University of Colorado Institutional Animal Care and Use Committee has approved the animal protocol (no. 00084) for this research.

### Encapsulation of colonies into allyl sulfide hydrogels

Organoids were released from Matrigel using mechanical stimulation from a pipette tip with cold basal medium and centrifuged (at 900 rpm for 4 min) to form a pellet. The pelleted organoids were enzymatically dissociated into single cells by incubation at 37°C for 8 min in 1 ml of TrypLE (Life Technologies), supplemented with deoxyribonuclease I (~10 mg; Sigma-Aldrich), 1 mM *N*-acetylcysteine (Sigma-Aldrich), and 10 mM Y27632 (Stemgent). Following incubation, the single-cell suspension was diluted with 1 ml of fetal bovine serum and 3 ml of basal medium and passed through a 40-μm filter to remove multicellular aggregates. The single-cell suspension was then centrifuged (at 1200 rpm for 4 min), and the pellet was resuspended in Matrigel, which was subsequently plated at 10-μl drops onto a warmed 48-well plate. The cell laden Matrigel drops were allowed to gel for 10 min at 37°C, before the addition complete medium supplemented with thiazovivin (2.5 mM; Stemgent). From the plated single cells, intestinal colonies were allowed to form over the course of 3 days. After 3 days in culture, hydrogel precursor solutions were prepared by diluting PEG-8DBCO (40 kDa) to 4 wt % in basal medium. Azide-functionalized Arg-Gly-Asp (RGD) (0.8 mM) was added to the hydrogel precursor solutions and allowed to react at room temperature for 5 min before being placed on ice. Once chilled, laminin (0.1 mg/ml, source) was added to the hydrogel precursor solution and mixed vigorously. Three-day-old colonies were released from Matrigel using mechanical stimulation from a pipette tip with cold basal medium and centrifuged (at 900 rpm for 4 min) to form a pellet. The cell pellet was resuspended in basal medium and added to the hydrogel precursor solutions on ice. Gelation was initiated by the addition of allyl sulfide diazide, and the solution was immediately vortexed for 5 s and added as 6-μl drops sandwiched between thiol-functionalized coverslips and glass slides treated with Sigmacote (Sigma-Aldrich), separated by 250-μm spacers. The gels were left for 10 min to gel at room temperature and removed from the glass slides, and gels adhered to coverslips were placed wells with complete medium.

### Photopatterning of curvature change in encapsulated organoids

After 1 day after encapsulation, LAP (1 mM) was added to each well and allowed to equilibrate for 15 min. Individual gels attached to coverslips were removed from medium and inverted onto a circular rubber stand for microscopy. Photopatterning was performed on a laser scanning confocal microscope (Zeiss LSM 710). Using a pixel size of 290 nm, four ROIs of the appropriate width arranged around a colony were scanned in a single plane with the 405-nm laser (1 mW) at a laser power of 30% and a pixel dwell of 5.12 μs. Approximately 30 colonies per gel were patterned, and then, the hydrogel was placed in fresh complete medium. From images of patterned organoids, the Fiji plugin, Kappa, was used to trace by hand the epithelium within the patterned region to determine the epithelial curvature, κ, which was used to calculate the radius of curvature according to the following equation

$$\text{radius\textbackslash{} of\textbackslash{} curvature}=\frac{1}{\kappa }$$ (5)

### Differentiation of patterned organoids

Gels containing photopatterned organoids were equilibrated for 15 min with FluoroBrite medium (Thermo Fischer Scientific) containing Hepes, penicillin-streptomycin, and N2 and B27 supplements, along with glutathione (15 mM; Sigma-Aldrich), LAP (1 mM), and PEG_3_-azide (0.76 mM). Following equilibration, the medium was removed, and the gels were exposed to 365-nm light at 5 mW cm^−2^ for 40 s and immediately placed in complete medium without CHIR99021 and valproic acid (just ENR). The medium was replaced every 2 days.

### Analysis of crypt angle and orientation order parameter

Organoids were fixed using 4% PFA in PBS (for 30 min at room temperature) and imaged using a laser scanning confocal microscope (Zeiss LSM 710) to collect whole organoid *z*-stacks with transmitted light. Images were registered to align with the original photopattern, and the angle of crypt formation was manually measured using the line tool in Fiji (NIH). Crypt angles were separated into eight bins, corresponding to four photopatterned regions and four unpatterned regions of equal size. This binning strategy was chosen and adapted from Fedele *et al*. (*53*), in which four binning regions were each defined for the photopatterned and unpatterned regions. The calculation for the orientation order parameter was derived from Boeing (*30*). Briefly, the entropy of crypt orientations, *H*_o_, is given as

$$H_{o}=-\sum_{i=1}^{n}P(o_{i})\ln\;P(o_{i})$$ (6)

where *n* represents the number of bins, *i* indexes the bins, and *P*(*o_i_*) represents the proportion of crypts that fall within the *i*th bin. The orientation order parameter, ϕ, is calculated as

$$\phi =1-{\left(\frac{H_{o}-H_{g}}{H_{\max}-aH_{g}}\right)}^{2}$$ (7)

where *H*_max_ represents the maximum entropy, which is the natural logarithm of the number of bins (eight), while *H*_g_ represents the entropy of an idealized grid with all crypts falling within the four photopatterned regions and is equal to the natural logarithm of the number of photopatterned regions (four). Here, a ϕ value of 0 indicates low order (i.e., uniform distribution within all bins), while a ϕ value of 1 indicates high order (i.e., all crypts within an idealized number of bins and minimal possible entropy).

### FlipperTR tension probe FLIM imaging and analysis

FLIM measurements were taken on a Zeiss LSM780 (Carl Zeiss, Jena, Germany) confocal microscope with ZEN and ISS FastFLIM software and a titanium:sapphire Mai Tai HP (Spectra-Physics, Milpitas, CA) two-photon laser. FlipperTR tension probe experiments were performed at a concentration of 2 μM in FluoroBrite medium (incubating for at least 20 min before imaging) using 800-nm two-photon excitation, observed through a 40× water-immersion objective Zeiss Korr C-Apochromat numerical aperture of 1.2 (Carl Zeiss, Jena, Germany), isolated using a 488-nm longpass dichroic mirror (Semrock Di02-R488-25-D), and detected with a Hamamatsu H7422p-40 photon-counting photomultiplier tube (PMT) connected to a ISS A320 FastFLIM box (ISS, Champaign, IL); lifetime calibration was performed with an aqueous fluorescein calibration solution of 1 μM with a 4-ns lifetime. ISS Vista was used for analysis and lifetime fitting of frequency domain FLIM data. To compare patterned versus unpatterned regions, organoids patterned with four rectangles arrayed around a circle at 90° intervals were compared to unpatterned organoids. ROIs were selected of the organoid epithelium at each quadrant for individual thresholding and lifetime fitting so that each organoid resulted in four lifetime measurements (one in each patterned region), with matched values from unpatterned organoids. Gaussian smoothing was used for phasor analysis of FLIM data in ISS Vista.

### DiSBAC_2_ (3) resting membrane potential imaging and analysis

Shortly after patterning, organoids were incubated in a 2 μM solution of DiSBAC_2_ (3) (Thermo Fisher Scientific) in FluoroBrite medium for at least 20 min and imaged using confocal microscopy (Zeiss LSM 710). High-resolution *z*-stacks were acquired of live organoids (~1-μm slices), with images centered at the apex of epithelial deformation following photoinduced shape change. In Fiji, a sum slices projection was generated, and the freeform selection tool was used to manually segment patterned and unpatterned regions of epithelium. Fluorescence intensity in these regions was measured, and all values were normalized to the average of the unpatterned regions for each organoid. Representative images were made using the “thermal” lookup table in Fiji.

### Tat-GAP19 treatment

Organoids were incubated in medium with tat-GAP19 peptide and added at a final concentration of 50 μM for 4 to 6 hours after photopatterning for analysis of the resting membrane potential or 24 hours following photopatterning for analysis of YAP localization.

### YAP staining and analysis

Organoids were fixed (4% PFA in PBS for 30 min at room temperature) immediately or 24 hours after photopatterning. Fixed organoids were then solubilized using 0.2% Triton X-100 (Sigma-Aldrich) in PBS (1 hour at room temperature) and blocked using 10% horse serum (Gibco) and 0.01% Triton X-100 in PBS (overnight at 4°C). The samples were then incubated overnight at 4°C with a mouse primary antibody against YAP (1:100; Santa Cruz Biotechnology, sc-101199) diluted in blocking buffer. After washing with fresh PBS every hour for 5 hours to remove residual primary antibody, the samples were incubated with rhodamine phalloidin (1 U/ml; Invitrogen), DAPI (4′,6-diamidino-2-phenylindole; 1.5 μM), and an Alexa Fluor 647 goat anti-mouse (1:1000; Invitrogen, A21235) secondary antibody in blocking buffer. After washing with PBS to remove any residual antibody, the fluorescently labeled organoids were imaged using confocal microscopy (Zeiss LSM 710) to collect whole organoid *z*-stacks. To quantify YAP localization, Fiji was used to isolate outlines of nuclei from the DAPI channel, which were overlaid onto the YAP channel. Straight lines were drawn on this merged image to bisect each nucleus, passing through the apical and basal side of each cell. An intensity line scan was used to determine the fluorescent intensity along this path, where the demarcations for the nucleus were used to determine the nuclear versus cytoplasmic YAP intensity. The intensity values were used to calculate the nuclear:cytoplasmic ratio for cells within photopatterned and non–photopatterned regions.

### Statistical analysis

All statistical analyses were performed in Prism (GraphPad). To assess statistical significance, two-tailed, unpaired Student’s *t* test, one-way analysis of variance (ANOVA), and two-way ANOVAs were performed, and *P* < 0.05 was considered significant.
