# Supplementary material for: In situ modulation of intestinal organoid epithelial curvature through photoinduced viscoelasticity directs crypt morphogenesis
Source: Sci Adv. 2023 Jan 20;9(3):eadd5668. doi: 10.1126/sciadv.add5668 (PMC9858500; doi:10.1126/sciadv.add5668)
Supplement: Supplementary file 1 — Supplementary Text Figs. S1 to S9 [file sciadv.add5668_sm.pdf]

Science Advances

Supplementary Materials for

In situ modulation of intestinal organoid epithelial curvature through photoinduced viscoelasticity directs crypt morphogenesis

F. Max Yavitt *et al.*

Corresponding author:email: Kristi S. Anseth, kristi.anseth@colorado.edu

Sci. Adv. **9**, eadd5668 (2023)
DOI: 10.1126/sciadv.add5668

This PDF file includes:

Supplementary Text
Figs. S1 to S9

Supplementary Materials

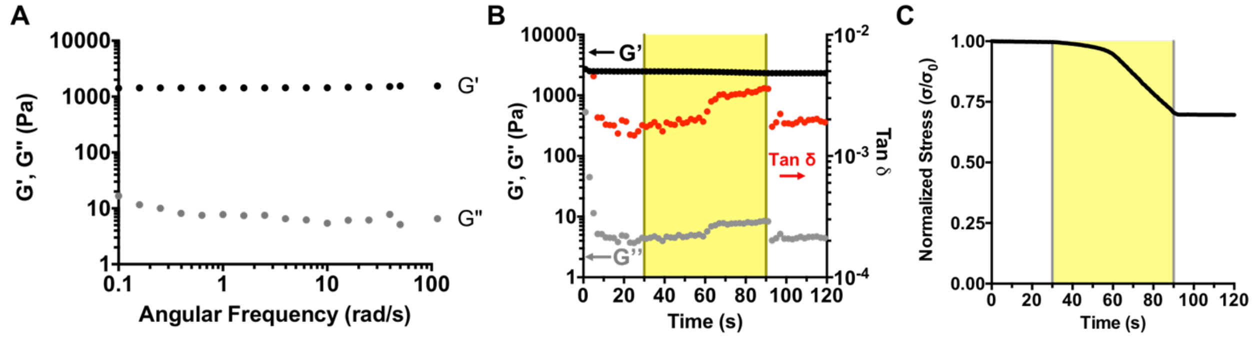

Supplementary Figure 1. Photoinduced viscoelastic response. **A** A frequency sweep of the shear storage (black) and loss (gray) moduli show a lack of frequency dependence in the absence of light, which is indicative of elastic networks. **B** The loss modulus of allyl sulfide hydrogels increased during irradiation (yellow, 10mW cm^{-2}, 405nm light), and decreased upon shuttering of the light. **C** The normalized stress decreased during irradiation (yellow, 10mW cm^{-2}, 405nm light). All plots show a representative trace.

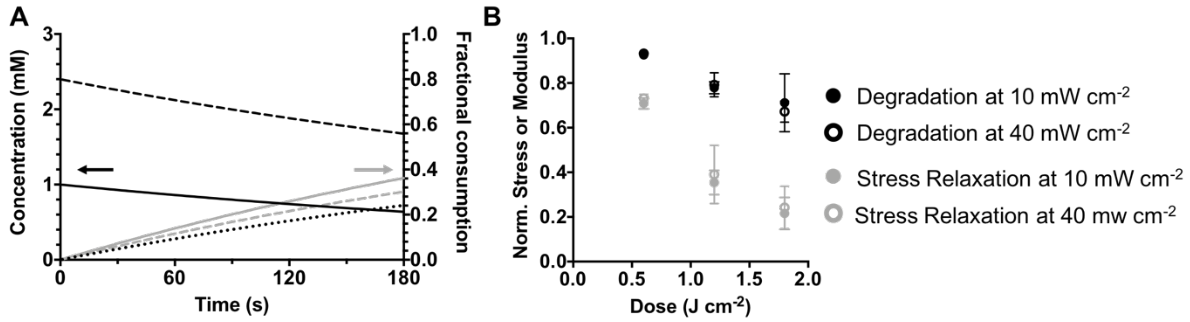

Supplementary Figure 2. Allyl sulfide and LAP concentration and consumption. A The concentration (left axis) of LAP (black, solid) decreases and the concentration of radicals (black, dotted) increases as LAP undergoes photolysis during irradiation with light (10 mW cm^{-2}, 405 nm, t = 0 seconds). Meanwhile, the concentration of allyl sulfide groups (black, dashed) decreases as LAP radicals react with allyl sulfide groups through irreversible cleavage reactions. The fractional consumption (right axis) of LAP (gray, solid) and allyl sulfide groups (gray, dashed) increases during the same irradiation. **B** Degradation (black) and stress relaxation (gray) is dependent on the total light dose for light intensities of 10 mW cm^{-2} (closed circles) and 40 mW cm^{-2} (open circles).

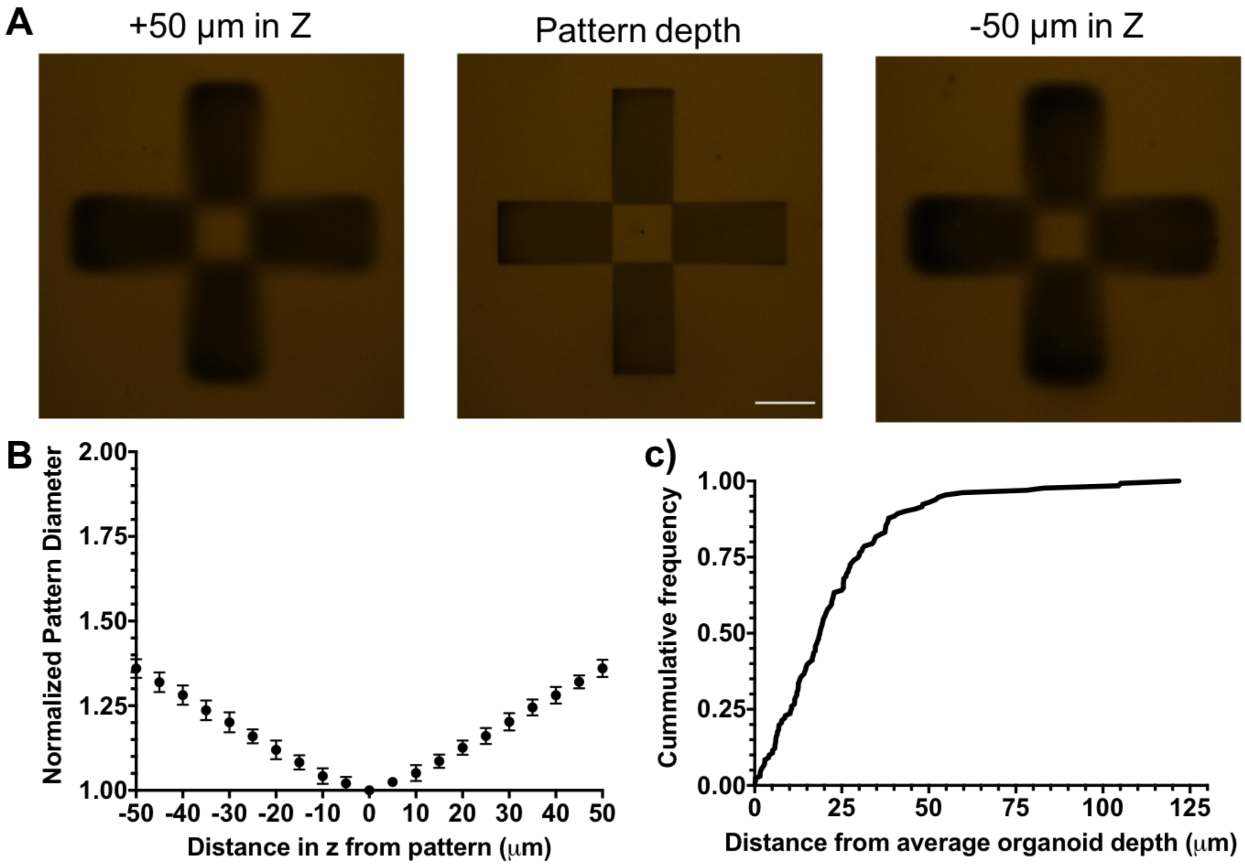

Supplementary Figure 3. Depth and height response to photopatterning. A A fluorophore that is quenched in the presence of radicals was included into the hydrogel network (orange). A cross was subsequently patterned at a single z plane to visualize the pattern dimensions 50 μm above (left) and below (right) of the focal plane (middle). Scale bar is 50 μm. **B** The pattern diameter increases above and below the focal plane due to excitation from out of focus light. **C** The cumulative frequency of organoid distribution within the z dimension of the hydrogel shows that most organoids are within a 50 μm range in z depth.

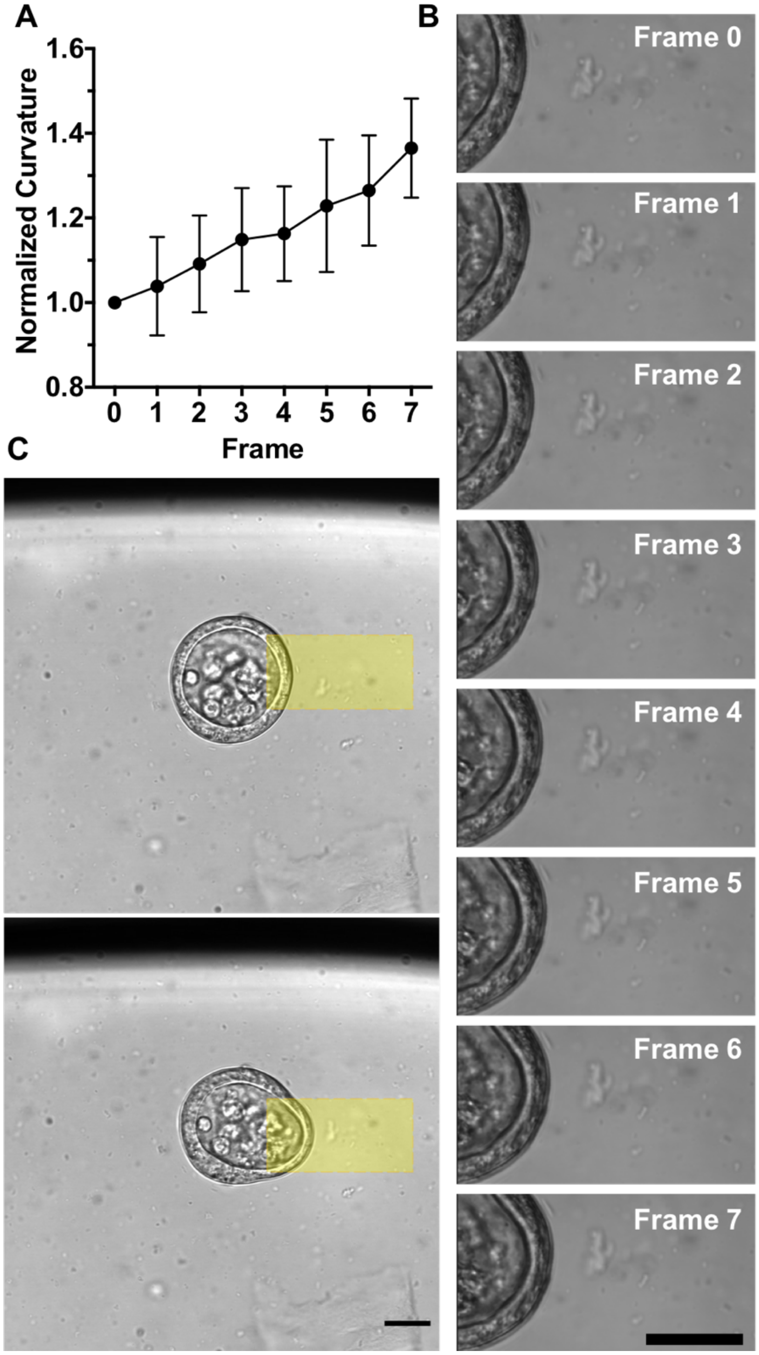

Supplementary Figure 4. Sequential deformation of organoid photopatterning. A The photopatterning method used requires 8 sequential passes over the photopatterned region. An increase in the normalized curvature is seen after each successive pass, highlighting the immediate response of the organoid to the photopatterned reaction. **B** The organoid curvature can be seen increasing over each successive frame. Scale bar is 30 μm. **C** The curvature of the pre and post patterning states can be visualized. The yellow box highlights the photopatterned area. Scale bar is 30 μm.

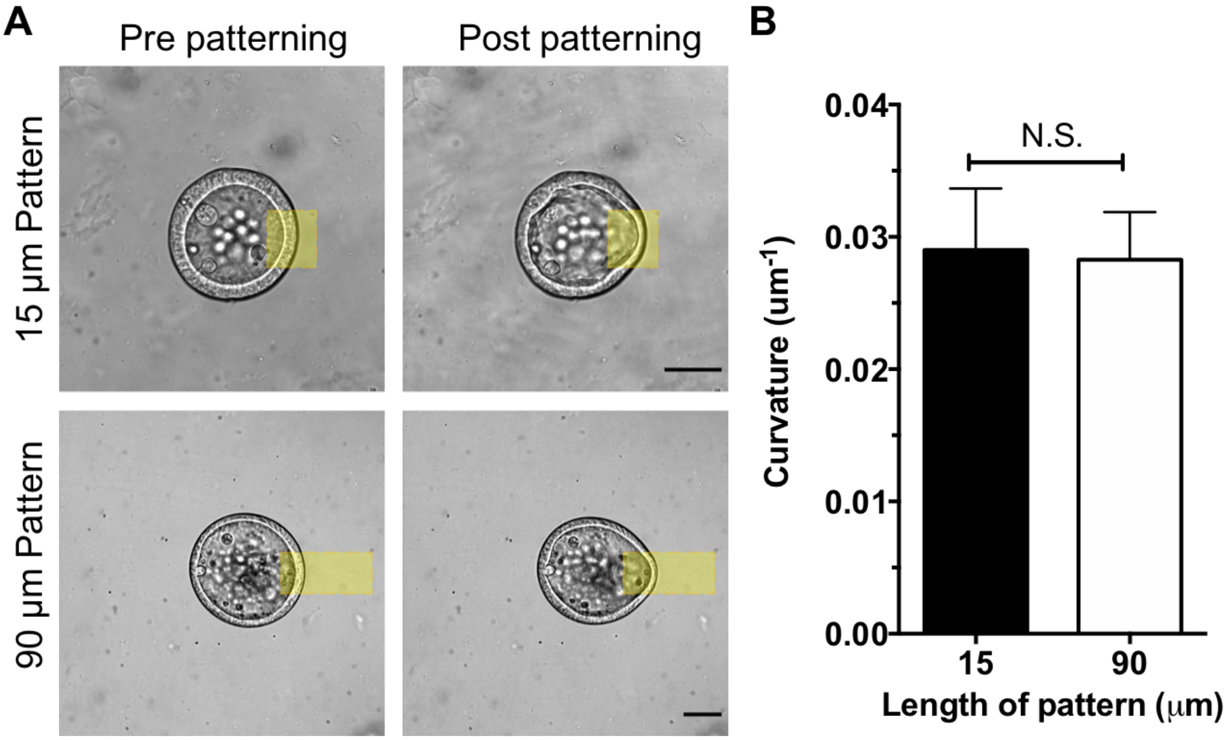

Supplementary Figure 5. Pattern length. A Photopatterns 15 µm and 90 µm in length were applied to encapsulated organoids, and images were collected soon after patterning. Scale bar is 50 µm. **B** The epithelial curvature determined immediately after patterning is similar for the 15 and 90 µm patterns. Significance determined by t-test, N = 15 organoids per condition, mean ± standard deviation.

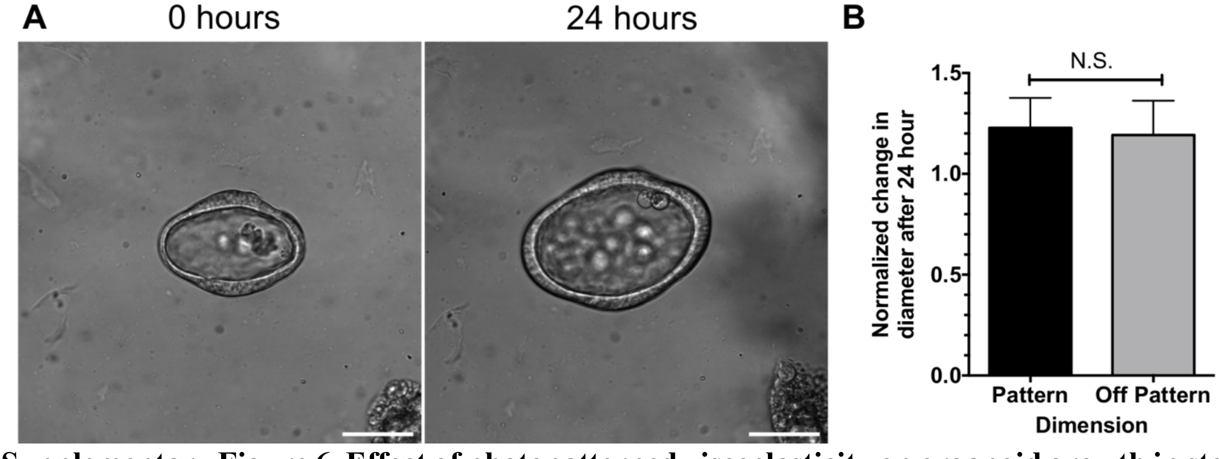

Supplementary Figure 6. Effect of photopatterned viscoelasticity on organoid growth in stem conditions. **A** Organoids were imaged 0 hours and 24 hours after patterning with 50 µm patterns on opposite sides (0° and 180° on the unit circle). Scale bar is 50 µm. **B** The normalized change in diameter between the 0 and 24 hour conditions does not change in the patterned (0° and 180° on the unit circle) and off-patterned (90° and 270° on the unit circle) dimensions. Significance determined by t-test, N = 15 organoids.

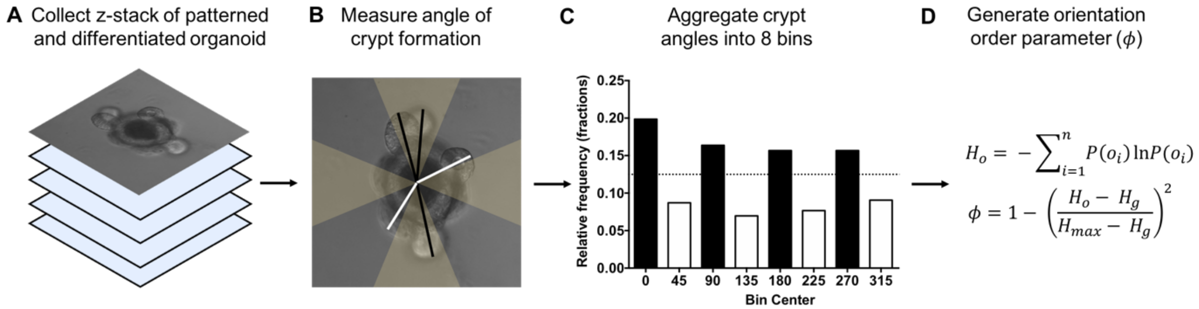

Supplementary Figure 7. Determination of crypt orientation order parameter. **A** Organoids were fixed and imaged to collect whole organoid z-stacks with transmitted light. **B** The angles of crypt formation for all crypts were determined manually and highlighted according to the position of the original photopatterned regions (yellow sections). Crypts originating from the original photopatterned regions are denoted with black lines, while crypts outside of the original photopatterned regions are denoted with white lines. **C** The crypt angles were aggregated and binned according to the original photopatterned regions, where black bars denote crypts that originated within photopatterned regions, while white bars denote crypts that did not originate from photopatterned regions. The dotted line marks the relative frequency if all crypts were equally distributed among the bins. **D** The relative frequencies were used to generate the orientation order parameter according to Boeing et al. (30). An orientation order parameter of 0 indicates that the system is entirely disordered, with an even distribution of crypt angles across all bins, while an orientation parameter of 1 indicates that the system is perfectly ordered, with all crypts aligned within a specific number of bins.

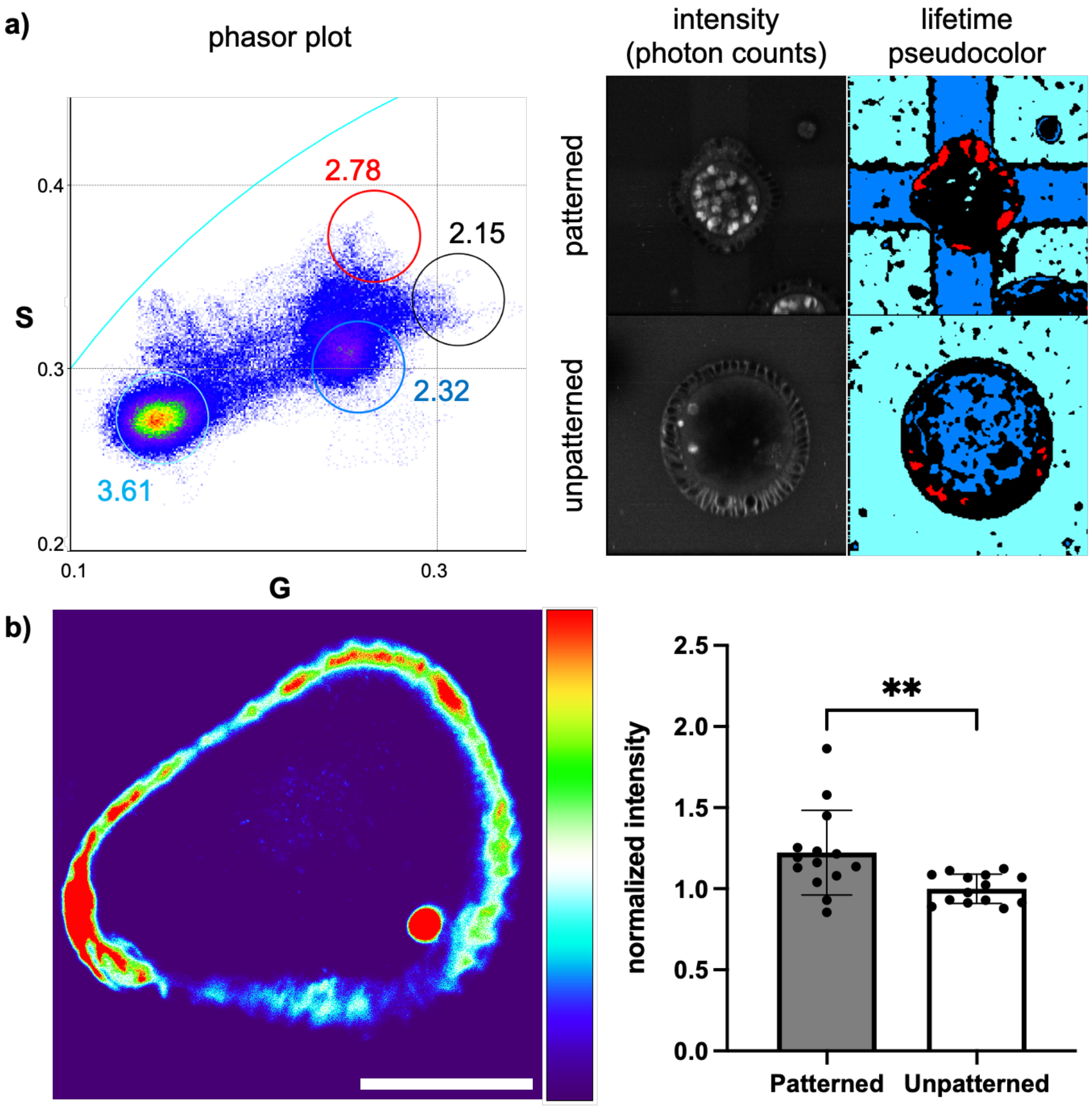

Supplementary Figure 8. FLIM and DiSBAC$_2$(3) analysis of patterned organoids. a) Phasor approach to lifetime analysis of FlipperTR membrane tension probe (τ_φ labeled on plot) reveals a ~0.6 ns increase in tension in the membrane of organoids with photoinduced shape change (black and red circles in pseudocolor). Incidentally, allyl sulfide hydrogels exhibit a striking reduction (1.3 ns) in background fluorescence lifetime after patterning (light vs. dark blue circles in pseudocolor), which is likely related to the presence of tethered LAP fragments in the network following irradiation. **b)** Alternative organoid geometries (e.g., a 90° angle) maintain localization of increased membrane depolarization in patterned regions of increased curvature. Scale bar is 50 µm. Significance determined by unpaired t-test with Welch's correction. N = 14 paired patterned vs. unpatterned regions from 7 organoids, mean ± standard deviation.

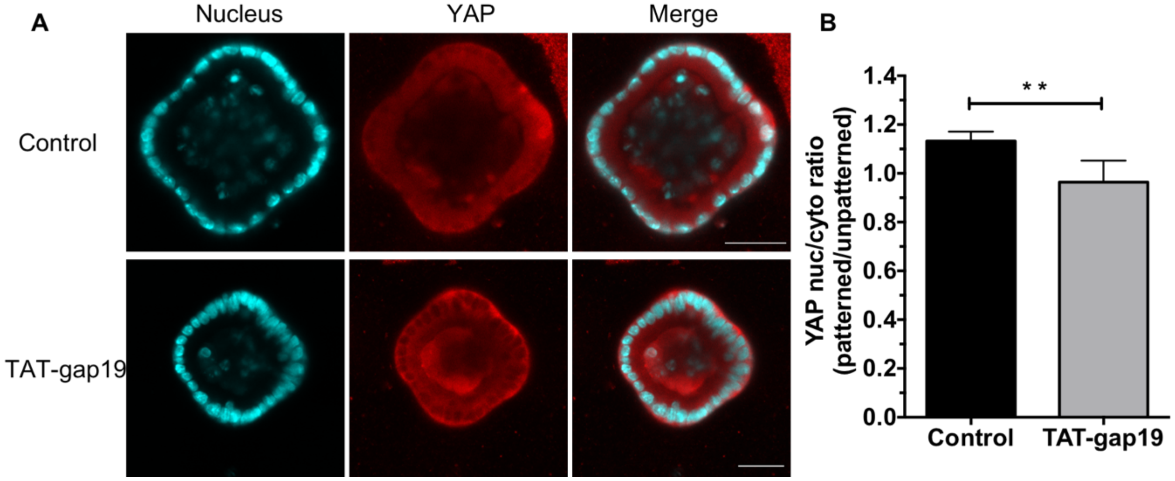

Supplementary Figure 9. **TAT-gap19 inhibits YAP nuclear localization caused by patterning epithelial shape**. **A** Nuclei (cyan) and YAP (red) shown 24 hours after photopatterning in organoids. Scale bar is 50 µm. **B** Treatment with TAT-gap19 lowers the YAP nuclear to cytoplasmic ratio, normalized between patterned and unpatterned regions. Significance determined by t-test with Welch's correction. Data shown is the average and standard deviation of N = 5 organoids (57 cells in patterned and 48 cells in unpatterned regions) for the control condition and N = 7 organoids (116 cells in patterned and 101 cells in unpatterned regions) for the TAT-gap19 treated condition.
